# Reducing the Time to Action on Bilirubin Results Overnight in a Newborn Nursery

**DOI:** 10.1097/pq9.0000000000000707

**Published:** 2023-12-12

**Authors:** Andrew M. Beverstock, Lily Rubin, Meredith Akerman, Estela Noyola

**Affiliations:** From the * Department of Pediatrics, NYU Langone Hospital Long Island, Mineola, New York; † Biostatistics Core, Division of Health Services Research, NYU Long Island School of Medicine, Mineola, New York.

## Abstract

**Introduction:**

Infants commonly require phototherapy in the nursery to prevent kernicterus, but it can interfere with parent-infant bonding. Minimizing unnecessary phototherapy is important. We noticed frequent delays in initiating and discontinuing phototherapy at our hospital. Our primary aim was to start or stop phototherapy within 3 hours of the intended blood draw time for more than 80% of patients by August 2022. Our secondary aims were to have the bilirubin result available within two hours of the intended draw time and for the result to be actioned upon within 1 hour of becoming available.

**Methods:**

We audited all patients requiring phototherapy, from January 2021 to December 2021 (n = 250). In PDSA cycle 1, we used electronic medical record result alerts. In cycle 2, we educated residents on the importance of acting promptly on results. In cycle 3, we asked residents to message the nurse to alert them to any laboratory draws for that shift. In cycle 4, we implemented a standardized laboratory draw policy.

**Results:**

We increased the percentage of results acted upon within 3 hours from 56% to more than 80%. We also reduced the mean time from blood draw to action from 184 minutes to 134 minutes. The time from intended draw to result availability decreased from 115 minutes to 95 minutes, and the time to action decreased from 67 minutes to 42 minutes.

**Conclusions:**

Combining resident education, electronic medical record result alerts, and policy standardization allowed us to achieve our stated aim and improved care for our neonates.

## INTRODUCTION

### Problem Description

Infants commonly require blue light phototherapy in the newborn nursery due to elevated unconjugated bilirubin levels. The American Academy of Pediatrics guidelines contain nomograms indicating the threshold for initiating phototherapy based on an infant’s gestational age and the presence or absence of neurotoxicity risk factors.^1^ The 2022 revised guidelines^2^ adjusted the phototherapy thresholds and provided guidance for pediatric practitioners about when to initiate and discontinue phototherapy. Starting phototherapy promptly is vital for preventing serum bilirubin levels, which may put infants at risk of permanent damage from acute bilirubin encephalopathy and kernicterus. At our center, we noted frequent delays in initiating and discontinuing phototherapy during resident night shifts in our well-baby nursery, exposing infants to these risks.

### Available Knowledge

While phototherapy is broadly safe, and the benefits of preventing acute bilirubin encephalopathy and kernicterus unambiguously outweigh the harms, recent research highlights phototherapy risks.^3–5^ Phototherapy utilization interferes with infant–mother bonding and breastfeeding, as it requires physical separation of the infant into an isolette or bassinet, which can interfere with breastfeeding.^6^ Phototherapy use may also be associated with a slight, yet significant increase in the risk of childhood epilepsy and hematologic malignancies. Therefore, avoiding unnecessary phototherapy is critical.

### Rationale

After noticing delays in starting and stopping phototherapy we decided to use quality improvement methodology to address the issue. Although the risks of phototherapy are small for each patient, it is such a commonly needed treatment that the total number of infants exposed to these risks is high. The first few days of life are crucial for establishing successful breastfeeding. It is, therefore, imperative to reduce disruptions to this, including separation of the infant from the mother for phototherapy.

We felt that reduced overnight staffing and, thus increased workload of phlebotomy, medical, and nursing staff may have contributed to delays in drawing and acting upon results and targeted interventions to address this by improving communication. Additionally, there is a large volume of published research showing a decreased cognitive performance by workers on night shifts.^7,8^ Consequently, we looked for interventions that would reduce the cognitive burden by using “result alerts” instead of requiring repeated checks.

### Specific Aims

We designed a quality improvement initiative to reduce these delays. Our primary measure was the time taken to start or stop phototherapy from the intended bilirubin lab draw time: we aimed to have more than 80% of results actioned upon within 3 hours of the planned draw time within 8 months of the start of the project (specifically by August 2022). We had two process measures: the time from the intended draw time to result availability in the electronic medical record (EMR) and the time from result availability to action. This was defined as either starting or stopping phototherapy.

## METHODS

This was a single-center quality improvement initiative and follows the Standards for Quality Improvement Reporting Excellence reporting guidelines.^9^

### Context

Our center is a large suburban hospital associated with a medical school. Our well-baby nursery has 48 beds, with 5161 deliveries per year, of which 12.6% are admitted to our level III NICU. Phlebotomists cover the entire hospital at night and can be paged to come on an “as needed” basis but do not routinely round on any particular floor at night. Depending on staffing availability, each mother-baby nurse has three or four mother-infant dyads at a time.

During the day, the nursery is covered by one pediatric resident and one attending: they leave at 5 pm and hand over to the NICU night resident. This resident covers the NICU (including rounding, attends deliveries), follows up daytime tasks from the well-baby nursery, and is supported by an at-home attending. The NICU resident is responsible for following up on bilirubin results until 7 am. They can be contacted either by pager or via Epic chat, but before our project, nurses were not able to check which resident was on call each night. A single resident covers the unit Monday through Friday nights for 2 weeks, with another resident covering the unit on Saturday and Sunday nights.

During the day, bilirubin levels are drawn by phlebotomy at set times (typically 7 am), and the results are followed by the daytime resident. During the night shift, for infants on the first day of life, the timing of these laboratories often depends on when an infant turns 24 hours old, as they are frequently drawn with the newborn blood spot screening test to avoid repeated blood draws. For infants older than 24 hours, blood draws are ordered at varying times throughout the night, based on the discretion of the ordering attending without any standardized timing.

In 2022, 8% of infants admitted to the well-baby nursery were treated with phototherapy. Our center implemented the 2022 AAP guideline recommendations in October of 2022, which likely led to a slight reduction in the percentage of infants requiring phototherapy compared with previous years.

### Interventions

**Fig. 1.**
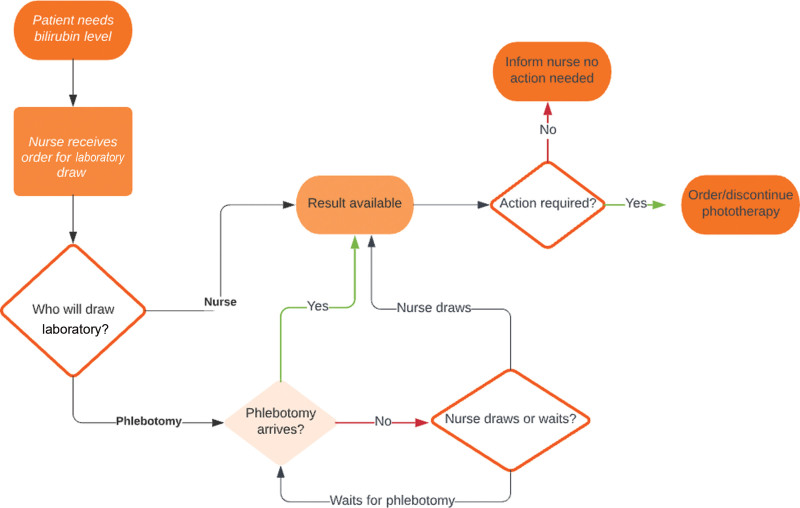
Process map for workflow of bilirubin draws and phototherapy orders.

In December 2021, we created a multidisciplinary quality improvement team consisting of newborn nursery nurse managers, pediatric residents, and a pediatric hospitalist attending. We identified neonates in our nursery who required phototherapy in the preceding 6 months, using a report on the Epic EMR (Epic Systems, Madison, Wis.) and conducted a retrospective chart review to obtain a baseline data set. We looked at all bilirubin results drawn between 5 pm and 7 am every day. Our team mapped the processes involved in acting on a bilirubin level (Fig. 1).

**Fig. 2.**
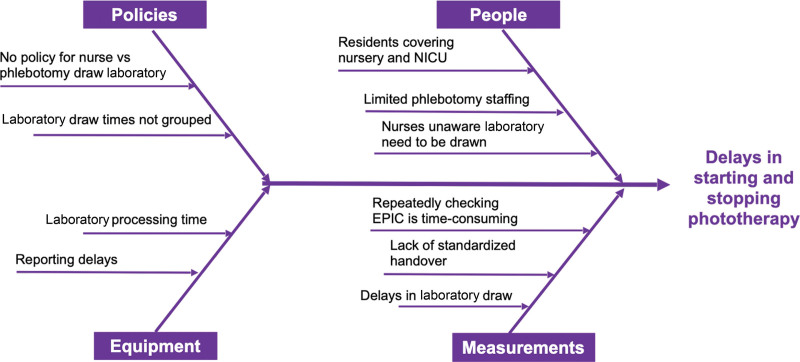
Fishbone diagram.

**Fig. 3.**
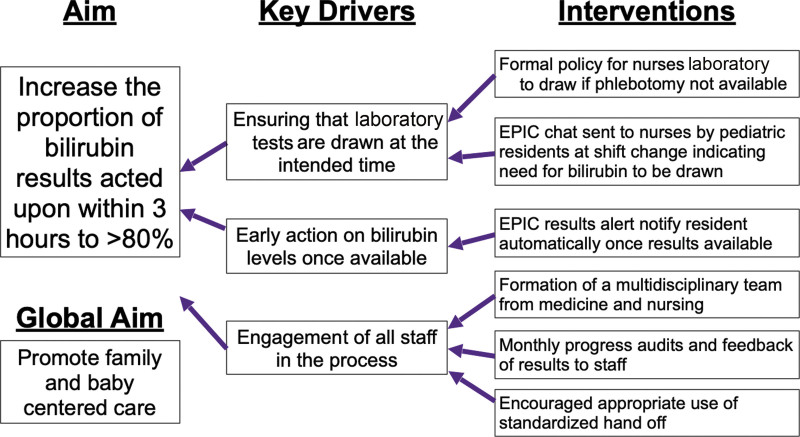
Key Driver Diagram.

We then created a fishbone diagram to look for factors that may contribute to delays (Fig. 2). We created a key driver diagram to help us design appropriate interventions for each possible delay (Fig. 3).

We conducted four PDSA cycles to achieve our target and re-audited each month (n = 331). In PDSA cycle 1 in January 2022, we surveyed pediatric residents about reasons for delays: all respondents indicated that they had sometimes been too busy with other tasks to check results promptly. We taught the residents how to set an Epic EMR “alert” that notified their when the bilirubin result was available. This alert was provided using the HIPAA compliant Epic app “Haiku.”

In PDSA cycle 2 in February 2022, we educated pediatric residents about promptly acting upon results. This was delivered as two lectures in an academic half-day lasting approximately 30 minutes: the lecture covered the risks of unnecessary phototherapy, reminded residents of how to set the Epic EMR “alert” and reviewed the AAP phototherapy guidelines. Around two-thirds of residents attended this lecture, and the lecture notes were available on a shared drive. The team also messaged the residents on NICU nights at the start of each block to remind them of our requested interventions.

In PDSA cycle 3 in April 2022, we advised residents to message the patient’s nurse at the start of their shift to inform them of any pending bilirubin levels to be drawn during the resident night shift to make sure that the laboratory labels were printed in time for phlebotomy’s arrival.

In PDSA cycle 4 in July 2022, we implemented a new policy in the nursery, where the infant’s nurse would draw a bilirubin level themselves if the phlebotomist had not collected it within 90 minutes of the intended time. This threshold was chosen based on discussion with both nursing leadership and phlebotomy. We repeated our resident education in November 2022 for our new class, before the new residents started NICU nights. Between each cycle we performed chart audits and calculated the proportion meeting our audit standard to evaluate their efficacy.

### Analysis

Results were inputted into Excel per institutional data storage policies, and a p-chart and X-bar charts were created from the data. These were analyzed using Nelson rule 2, looking for 8 points on the same side as the baseline mean indicating a significant change.

### Ethical Considerations

This study was deemed IRB exempt by our institutional review board.

## RESULTS

**Fig. 4.**
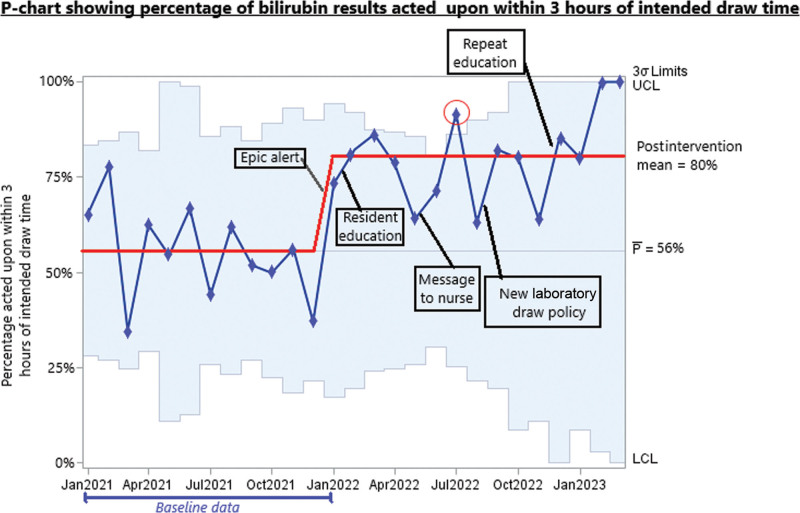
Percentage of bilirubin results acted upon within 3 hours of intended draw time, displayed on a statistical process control P-chart.

Our primary outcome is displayed in the P-chart (Fig. 4). It shows the overall improvement in the percentage of results acted upon within 3 hours, which rose from a baseline mean of 56% to a postintervention mean of 80%. The chart shows that at least 8 points were on the same side of the basline median, indicating a significant improvement in our outcome.

**Fig. 5.**
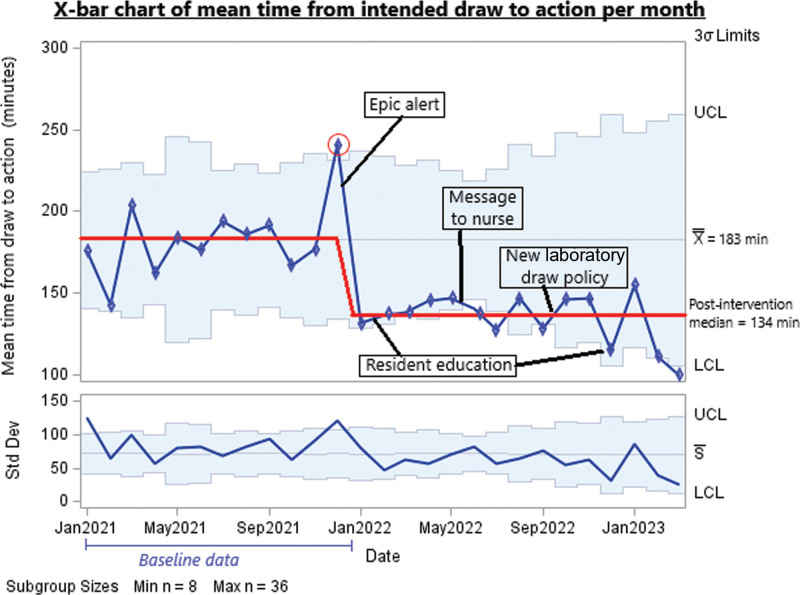
Time for entire process from intended draw to action displayed on X-bar chart.

Figure 5 shows a combination of our two process measure as an X-bar chart for the entire time from planned draw to action. The mean time from draw to action decreased from 184 minutes to 134 minutes, with a 49 minute average improvement in response time per patient.

**Fig. 6.**
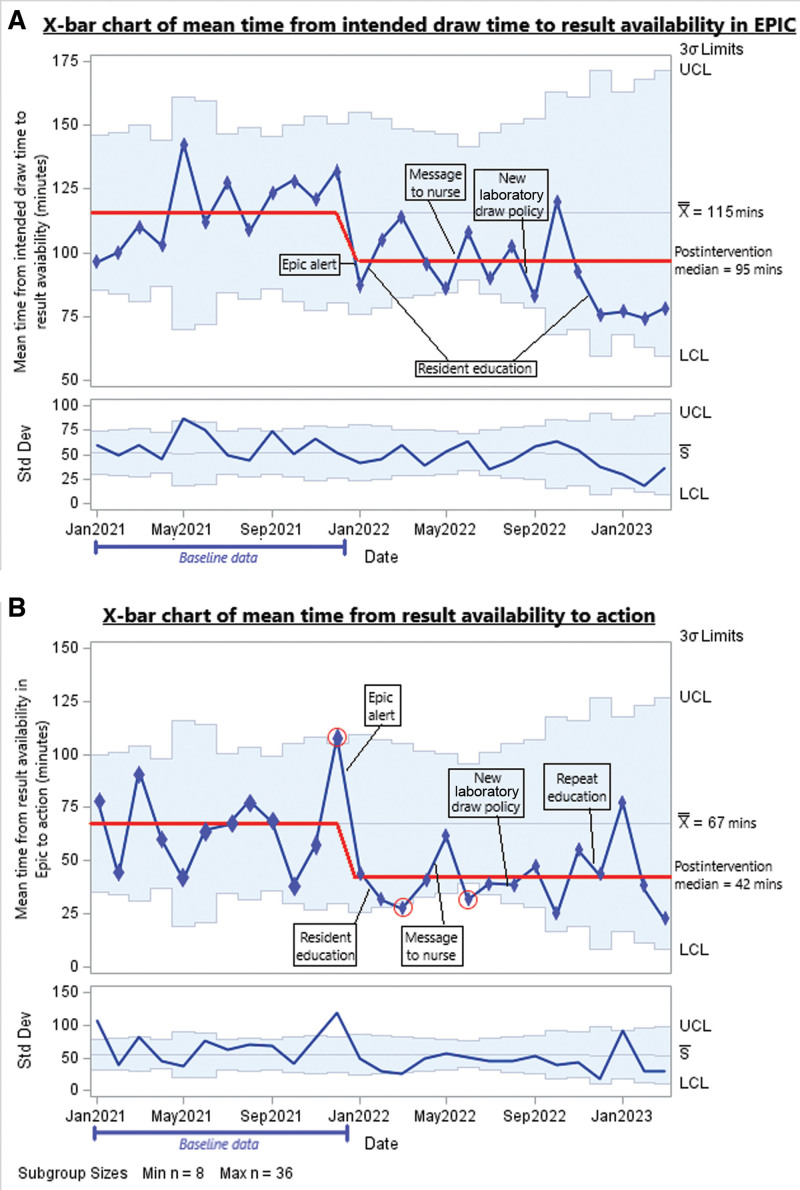
Process measure X-bar chart. A, Chart for intended draw to result availability. B, Chart for result availability to action.

Figure 6 shows an X-bar chart with our process measures separated into the draw and action phases. Both of these process measures improved. The mean time from intended draw to result availability decreased from 118 minutes in our baseline data to 75 minutes, and the time to action on a result once available decreased from 65 minutes to 41 minutes. Both indicate a significant improvement (8 points below the previous centerline).

There were no measured unexpected benefits or problems identified during this project. There was no change in staffing levels during the project, so there was no cost associated with the intervention.

## DISCUSSION

Our project was successful in ensuring that more than 80% of bilirubin results were acted upon within 3 hours, but we did not achieve our goal consistently by our intended target date of August 2022. Because we separately measured the time from intended draw to action and the time from result to action, we were able to target our interventions in each PDSA cycle to minimize the delay. Continuously collecting data at each step of the process and plotting it on a statistical process control chart allowed us to track our progress and target interventions appropriately. We were able to shift our mean from 56% in the baseline period to 80% during our interventions.

We found that delays in laboratory draws were preventing us from consistently achieving our target. To address this, we developed a standardized policy with nursing management where the infant’s nurse would draw the lab if the phlebotomy team did not arrive within 90 minutes of the intended time. We could implement these changes with relative rapidity because we had multidisciplinary involvement early in the project. Having a representative from phlebotomy on our team from the start of the project would likely have made this easier, as it may have reduced time spent meeting with different departments. Although the initial improvement was due to the use of the automated alert, standardizing laboratory draw times likely helped us sustain our positive change.

Figure 4 shows special cause variation for July 2022. We speculate that the special cause variation in this case may be because of an “early adopter” resident who used these interventions effectively leading to a particularly successful month.

Figures 5 and 6 show our process measures: Figure 5 shows the mean time from intended draw to action per month. The mean time decreased. All postintervention data points are on the same side of the mean, indicating a significant improvement (8 points above/below the previous centerline). One data point (December 2021) showed special cause variation above our upper control limit: the exact reason for this is not clear, but it is possible that high COVID-19 cases that year led to more staffing shortages than in other months and, thus a higher workload for staff. Four data points in the postintervention period were below our lower control limit, three during the summer. This may reflect the increased experience of residents toward the end of the academic year, as the June 2022 result was driven by a particularly short time from result availability to action.

This project used preexisting tools within the Epic EMR to achieve success. Previous work has looked at using abnormal result alerts in Epic to alert clinicians to abnormal results.^10,11^ This work found that it was an effective tool, but the alert was not a guarantee that the result would be acted upon. This previous work has focused on result flags in an outpatient setting, and no studies have been done looking at the use of result alerts for all inpatient results including “normal” in-range values. Even in-range values are important for pediatricians as they are used as a guide for when to stop phototherapy. The result alert function is available to all Epic users, but many are unaware of how to use it. This tool is particularly effective in a process requiring overnight follow-up of results. As the laboratory results can post at any time, it is very time-consuming and inefficient for the resident to keep checking result availability. An automated alert shows the responsible resident when the result is available and thus decreases time spent checking on results that may not be back. It also allows immediate action. We endorse the routine use of this tool in similar situations. Our project highlights the effectiveness of this tool to flag results for providers and allowing prompt action, instead of requiring providers to repeatedly check whether a result is available.

We plan to continue our project to maintain our success. Our next intervention will be to standardize draw times for bilirubin levels to ensure that laboratories can be batched by the phlebotomy team. This should further reduce delays in action.

Our project has several limitations. We only looked at instances where phototherapy was stopped or started as the time of order is recorded in Epic. Firstly, we were not able to collect data from instances where the bilirubin was still above threshold and the patient was already on phototherapy, or when they were below threshold but not on phototherapy, as nonaction is not recorded in Epic. In some cases residents may have decided to continue phototherapy, but we had no way to record the exact time of this in the EMR, and so we were unable to measure in these cases. Given that the resident did not know in advance whether an action would be taken or not, we felt that these studied situations would provide a realistic sample of when results were reviewed. We were also unable to directly check that residents were using the Epic alert system and messaging the nurse; however, the significant improvement in our results implies that our interventions were successful.

Secondly, our processes may not apply to larger centers with dedicated phlebotomy teams for the well-baby nursery and standardized laboratory draw times. However, all inpatient units have results that need to be followed overnight, and using Epic alerts can be a helpful tool for any situation where one provider must follow results with an unreliable resulting time.

Finally, we did not have a specific patient-facing outcome measure for the project, as there is no feasible way to measure mother–infant bonding on an objective basis, and the majority of patients follow-up with private pediatricians, so we have no way to follow-up long-term rates of breastfeeding or epilepsy. There were no balancing measures for this project.

In conclusion, through resident education, built-in EMR alert tools, and staff engagement, we successfully achieved our aim. We highlight the importance of a team-based approach and frequent data collection at each stage of the process to allow targeted interventions and continuous improvement throughout a quality improvement project.

## ACKNOWLEDGMENTS

We thank Dr. Ulka Kothari for her assistance in generating the Epic report that we used to identify patients for our audits.
